# The SNAP-*tag* technology revised: an effective *chemo-enzymatic approach* by using a universal azide-based substrate

**DOI:** 10.1080/14756366.2020.1841182

**Published:** 2020-10-29

**Authors:** Rosa Merlo, Diego Caprioglio, Michele Cillo, Anna Valenti, Rosanna Mattossovich, Castrese Morrone, Alberto Massarotti, Franca Rossi, Riccardo Miggiano, Antonio Leonardi, Alberto Minassi, Giuseppe Perugino

**Affiliations:** a Institute of Biosciences and BioResources, National Research Council of Italy, Naples, Italy; b Department of Pharmaceutical Sciences, University of Piemonte Orientale, Novara, Italy; c Department of Molecular Medicine and Medical Biotechnology, University of Naples “Federico II”, Naples, Italy; d IXTAL srl, Novara, Italy

**Keywords:** *Protein-tag*, protein labelling, enzymatic reaction, click chemistry, biotechnology

## Abstract

SNAP-*tag*^®^ is a powerful technology for the labelling of protein/enzymes by using benzyl-guanine (BG) derivatives as substrates. Although commercially available or ad hoc produced, their synthesis and purification are necessary, increasing time and costs. To address this limitation, here we suggest a revision of this methodology, by performing a *chemo-enzymatic approach*, by using a BG-substrate containing an azide group appropriately distanced by a spacer from the benzyl ring. The SNAP-*tag*^®^ and its relative thermostable version (*Ss*OGT-*H^5^*) proved to be very active on this substrate. The stability of these *tags* upon enzymatic reaction makes possible the exposition to the solvent of the azide-moiety linked to the catalytic cysteine, compatible for the subsequent conjugation with DBCO-derivatives by azide-alkyne Huisgen cycloaddition. Our studies propose a strengthening and an improvement in terms of biotechnological applications for this self-labelling *protein-tag*.

## 1. Introduction

The advent of the self-labelling *protein-tags* (SLPs) has led to a huge push in modern biotechnology, especially in the field of cell biology, where auto-fluorescent proteins (AFPs) for a long time dominated for their versatility in the localisation experiments of proteins, organelles, and membranes^1^. But the use of SLPs clearly goes beyond: they catalyse the covalent, highly specific and irreversible attachment of a part of their synthetic ligands upon reaction. This offers the opportunity to label them by conjugation to those ligands of an infinite number of chemical groups, such as fluorescent dyes, affinity molecules, or solid surfaces, expanding the application fields^2^. Among SLPs, of particular note are the *Halotag*^®^, the *SpyTag*^3^ the SNAP- and the CLIP-*tag*^®^. The Promega *Halotag*^®^ is a halo-alkane dehalogenase with a genetically modified active site, which reacts irreversibly with primary alkyl-halides^4^^,^^5^.

SNAP-*tag*^®^ from New England Biolabs (NEB) is the engineered variant of the natural suicide human *O^6^*-methylguanine DNA-methyltransferase protein (hMGMT). Alkylated DNA-alkyl-transferases (AGTs, MGMTs or OGTs, E.C. 2.1.1.63) are ubiquitous and conserved proteins involved in the repair of the DNA alkylation damage, in particular, they remove alkyl adducts at the level of *O^6^*-position on guanine base^6^^,^^7^. The peculiar single-step mechanism are called “*suicide enzymes*,” in which the alkylated base is directly repaired by the irreversible transfer of the alkylic group from the damaged guanine to the catalytic cysteine in the protein active site^8^. The protein is permanently inactivated upon the trans-alkylation reaction and susceptible to *in vivo* degradation via the proteasome.

**Figure 1.**
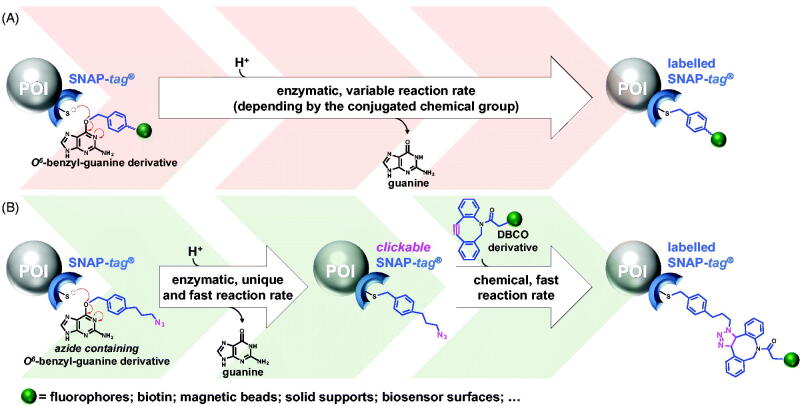
Single-step reaction vs chemo-enzymatic approach. (A) The SNAP-*tag*^®^ technology is based on BG-derivatives singularly synthetised and purified, and not excluding that the conjugated chemical group (*green sphere*) could affect the enzymatic reaction rate. (B) The SNAP-*tag*^®^ technology revised uses a unique and universal azide BG-derivative, converting SNAP-*tag*^®^ in a clickable form, prone to perform a fast and efficient cycloaddition with DBCO-based chemical groups. POI, protein of interest genetically fused to the SNAP-*tag*^®^.

In 2003, the group of Kai Johnsson developed a new strategy to exploit the hMGMT suicidal reaction in biotechnology, adopting a directed-evolution approach to engineer a variant to be used as an innovative *protein-tag*, that is, the SNAP-*tag*^®^. The rationale behind the SNAP-*tag* technology is the low substrate specificity of some AGT proteins, being able to efficiently recognise also the *O^6^*-benzyl-guanine (BG) nucleobase^9^. Likely, the reaction of these enzymes with BG-derivatives could happen: upon the irreversible transfer to the catalytic cysteine, they indeed demonstrated the specific labelling of the hMGMT with molecules, as fluorophores, previously conjugated to the 4-position of the BG benzyl ring. Because of the small dimension of this protein, it was mutagenized to abolish any DNA binding activity and utilised as *protein-tag* for the indirect labelling of proteins of interest genetically fused to it (Figure 1)^9–13^. Later, the same group further engineered the SNAP-*tag*^®^ to obtain the CLIP-*tag*^®^, which specifically reacts with *O^2^*-benzyl-cytosine derivatives, expanding that technology for *in vivo* and *in vitro* multi-protein labelling^14^.

Apart from cell biology and fluorescence imaging, hundreds of papers are present in the literature showing many applications of SNAP-*tag*^®^ in several fields, among which RNA-editing^15^, the development of SNAP-based sensors for small molecules^16–18^ and ions^19^^,^^20^, and protein-DNA complexes in “DNA Origami” structures^21^.

Following the same approach, Perugino and co-workers expanded this technology to extremophilic organisms and to all the applications which require harsh reaction conditions, not fully suitable for the employing of the mesophilic SNAP-*tag*^®^. To this aim, they developed a “*thermo*-SNAP-*tag*” by the production of a variant of the OGT from *Saccharolobus solfataricus* (previously *Sulfolobus solfataricus*, *Ss*OGT-*H^5^*, hereinafter *H^5^*), an enzyme which revealed extremely resistant to high temperature, high ionic strength, proteases attack, and, in general, to common physical and chemical denaturants^22^^,^^23^. The intrinsic stability of *H^5^* made it compatible with expression and utilisation *in vivo* as *protein-tag* in thermophilic organisms, as *Thermus thermophilus*^24^ and *Sulfolobus islandicus*^25^ as well as in an *in vitro* expression system using *Sulfolobus* lysates^26^. Recently, *H^5^* became a part of the new ASL*^tag^* system^27^, which was particularly useful for the *in vivo* immobilisation and contemporary labelling of proteins and enzymes of interest, stabilising them without any purification procedures needed^28^.

SNAP-*tag*^®^ technology is essentially based on BG-substrates: although many of them are commercially available, the possibility of conjugation of infinite desired molecules to the 4-position on BG leads to the synthesis of *ad hoc* substrates. This is generally possible through the crosslinking reaction of the so-called “BG-building block” (such as the amine-reactive BG-NH_2_) with NHS-ester derivative compounds. The main disadvantage is the need to purify the final compounds before the reaction with the enzyme, increasing the times and costs of the experiments (Figure 1(A)). Furthermore, the presence of chemical groups conjugated to the benzyl moiety of the BG could affect the reaction efficiency of the SNAP-*tag*^®^^29–33^, sometimes making this enzyme not fully applicable to particular requests.

In this work, we analysed and confirmed the catalytic dependence of SNAP-*tag*^®^ and *H^5^* by several substrates having different chemical groups conjugated to the *O^6^*-position of the guanine. To overcome these limitations, in the current study we suggest a further improvement of this technology with the application of a *chemo-enzymatic approach*, by using a unique and universal azide decorated BG-derivative, to obtain the specific labelling of the *tag* (*clickable*-SNAP), that can be easily coupled with a potentially infinite number of commercially available di-benzo-cyclo-octyl (DBCO)-based molecules, through the copper-free azide-alkyne Huisgen cycloaddition (Figure 1(B)). This approach could mainly offer the advantage to take into account of a unique reaction rate for the enzyme (with the azide-based BG), saving costs and times for the linking to the *tag* of an infinite number of commercially available DBCO-molecules. Here, we successfully proved the labelling of the SNAP-*tag*^®^ with several DBCO-based fluorophores and the covalent immobilisation of this protein on alkyne-coated surface sensors.

## 2. Materials and methods

### 2.1. Reagents

**BG** was from Activate Scientific GmbH (UK), whereas **MGPA** was a gift of Prof D. Prosperi (University of Bicocca, Milan, Italy). SNAP-Vista^®^ Green (**SVG**), SNAP Cell^®^ Block (**SCB**), SNAP Cell^®^ 430 (**SC430**), BG-PEG-NH_2_ (**BGPA**), pSNAP-*tag*(m) plasmid, DNA restriction endonucleases and DNA modification enzymes were purchased from New England Biolabs (USA). Molecular biology kits for plasmid preparations were from Macherey-Nagel GmbH (Germany). Oligonucleotides synthesis and DNA sequencing service were performed by Eurofins Genomics (Germany). **BDP FL alkyne, BDP FL DBCO**, **Cy5 DBCO** were purchased from Lumiprobe GmbH (Germany). **DBCO-PEG_4_-Fluor 545**, Tris(2-carboxyethyl)phosphin (TCEP), Tris [(1-benzyl-1H-1,2,3-triazol-4-yl)-methyl]amine (TBTA) were from Sigma-Aldrich (St. Louis, MO). Pierce™ Premium Grade Sulfo-NHS (N-hydroxy-sulfo-succinimide) and Pierce™ Premium Grade 1-ethyl-3-(3-dimethyl-amino-propyl)-carbodiimide hydrochloride (EDC) were from Thermo Fisher Scientific (Carlsbad, CA).

### 2.2. Compounds synthesis: general procedures

^1^H (400 MHz) and ^13^C (100 MHz) NMR spectra were measured on Bruker Advance Neo 400 MHz spectrometer. Chemical shifts were referenced to the residual solvent signal (CDCl_3_: *δ*_H_ = 7.26, *δ*_C_ = 77.0; DMSO: *δ*_H_ = 2.50 *δ*_C_ = 39.5). Low-resolution ESI-MS were obtained on an LTQ OrbitrapXL (Thermo Scientific) mass spectrometer. IR spectra were registered on Shimadzu DR 8001 spectrophotometer. Silica gel 60 (70–230 mesh) used for gravity column chromatography (CC) was purchased from Macherey–Nagel. Reactions were monitored by TLC on Merck 60 F254 (0.25 mm) plates, visualised by staining with 5% H_2_SO_4_ in ethanol or KMnO_4_ and heating. Organic phases were dried with Na_2_SO_4_ before evaporation. Chemical reagents and solvents were from Aldrich, Alfa Aesar, and TCI and were used without any further purification unless stated otherwise.

### 2.3. Synthesis of BGN3

**BGN3** was synthesised according to the method of Zhang et al.^34^, whose experimental spectra were comparable. White solid. ^1^H NMR (400 MHz, DMSO-d_6_) *δ* 7.84 (s, 1H), 7.53 (d, *J* = 7.8 Hz, 2H), 7.39 (d, *J* = 7.8 Hz, 2H), 6.28 (bs, 2H), 5.49 (s, 2H), 4.45 (s, 2H) (Figure S1(A)). ^13^C NMR (100 MHz, DMSO-d_6_) *δ* 159.69, 136.80, 135.43, 128.82, 128.58, 66.44, 53.37. IR (KBr) cm^−1^: 3638, 3462, 3322, 2799, 2132, 1424, 1257, 1163, 912, 790, 656, 514. ESI/MS: *m/z* [M + H^+^] 297 (Figure S1(B)).

### 2.4. Synthesis of BGSN3

**BGSN3** was synthesised by following the scheme in Figure S2.

#### 2.4.1 Synthesis of 4-azido-N-(4-(hydroxymethyl) benzyl) butanamide (compound 3)

A stirred solution of compound **1** (see Figure S2; 1.176 g, 9.115 mmol, 1 eq/mol) was prepared according to the method by Huang et al.^35^ in DCM (30 ml), compound **2** (1.500 g, 10.939 mmol, 1.2 eq/mol; prepared according to the method by Leng et al.^36^ and TEA (5.08 ml, 36.460 mmol, 4 eq/mol) were added. The mixture was stirred for 10 min at room temperature, then T3P (50% solution in EtOAc, 10.85 ml, 18.230 mmol, 2 eq/mol) was slowly added dropwise, and the stirred reaction was left overnight at room temperature until the complete conversion of the starting material (TLC: PE-EtOAc 4:6; Rf 1 = 0.47; Rf 3 = 0.16). The reaction was quenched by the addition of BRINE and extraction with DCM. After drying (Na_2_SO_4_) and evaporation, the residue was purified by gravity column chromatography on silica gel (gradient PE-EtOAc from 6:4 to 3:7) to afford compound **3** as a white solid (660 mg, 30%). ^1^H NMR (400 MHz, CDCl_3_) *δ* 7.28 (d, *J* = 8.0 Hz, 2H), 7.20 (d, *J* = 7.9 Hz, 2H), 6.36 (t, *J* = 5.6 Hz, 1H), 4.63 (s, 2H), 4.35 (d, *J* = 5.7 Hz, 2H), 3.31 (t, *J* = 6.6 Hz, 2H), 2.27 (t, *J* = 7.3 Hz, 2H), 1.89 (p, *J* = 6.9 Hz, 2H) (Figure S3(A)).^13^C NMR (100 MHz, CDCl_3_) *δ* 171.93, 140.30, 137.28, 127.69, 127.17, 64.53, 50.65, 43.19, 32.96, 24.69. IR (KBr) cm^−1^: 3276, 3055, 2921, 2880, 2103, 1635, 1540, 1418, 1257, 1015, 827, 747, 677, 553. ESI/MS: *m/z* [M + H^+^] 249 (Figure S3(B)).

#### 2.4.2. Synthesis of (N-(4-(((2-amino-9H-purin-6-yl)oxy)methyl)benzyl)-4-azidobutanamide) (BGSN3)

To a cooled solution (0 °C) of compound **3** (400 mg, 1.611 mmol, 1 eq/mol) in dry DMF (10 ml) in a dry flask under N_2_ atmosphere, NaH (60% dispersion in mineral oil, 202 mg, 5.059 mmol, 3.14 eq/mol) was slowly added. The mixture was stirred at 0 °C for 10 min, then DMAP (16 mg, 0.129 mmol, 0.08 eq/mol) and compound **4** (451 mg, 1.772 mmol, 1.1 eq/mol; prepared according to the method by Kindermann et al.^37^ were sequentially added. The reaction was then heated at room temperature and stirred for 4 h until the complete conversion of the starting material (TLC: DCM-MeOH 9:1; Rf 4 = 0.70; Rf BGSN= 0.55), then quenched by slow addition of BRINE and extraction with EtOAc. After drying (Na_2_SO_4_) and evaporation, the residue was purified by gravity column chromatography on silica gel (gradient DCM-MeOH from pure DCM to 20:1) to afford **BGSN3** as a white solid (413 mg, 67%). ^1^H NMR (400 MHz, DMSO-d_6_) *δ* 12.48 (bs, NH purine, 1H), 8.43 (t, *J* = 5.9 Hz, 1H), 7.85 (s, 1H), 7.49 (d, *J* = 7.7 Hz, 2H), 7.30 (d, *J* = 7.8 Hz, 2H), 6.31 (bs, NH2 purine 2H), 5.50 (s, 2H), 4.30 (d, *J* = 5.9 Hz, 2H), 3.36 (t, *J* = 6.8 Hz, 2H), 2.26 (t, *J* = 7.4 Hz, 2H), 1.81 (p, *J* = 7.1 Hz, 2H) (Figure S4(A)). ^13^C NMR (100 MHz, DMSO-d_6_) *δ* 171.38, 159.91, 159.69, 155.23, 139.48, 137.90, 135.31, 128.60, 127.34, 113.57, 66.59, 50.36, 41.95, 32.23, 24.58. IR (KBr) cm^− 1^: 3647, 3484, 3379, 3282, 2794, 2100, 1580, 1403, 1282, 1163, 938, 835, 789, 635, 553. ESI/MS: *m/z* [M + H^+^] 382 (Figure S4(B)).

### 2.5 Plasmids and protein purification

The cloning procedures in the pQE31 expression vector (Qiagen, Germany) were similar for both proteins. In particular, the pSNAP-*tag*(m) Vector was used as a template to amplify the DNA fragment relative to the SNAP-*tag*^®^ gene, by using QE_SNAP-Fwd/QE_SNAP-Rev oligonucleotides pairs (5′-ATGGCAGGATCCAA TGGACAAAGACTGCGAAATG-3′/5′-CTATCAAAGCTTAACCCAGCCCAGGCTTGCCCA G-3′; *BamH* I and *Hind* III sites, respectively, are underlined). Afterwards, the resulting fragment and the pQE31 vector were digested with *BamH* I and *Hind* III restriction enzymes and ligated, leading to the final pQE-SNAP plasmid. The final SNAP-*tag*^®^ protein was expressed with an extra N-terminal aminoacidic sequence, comprising a His_6_-*tag* (MRGSHHHHHHTDP-). The ligation mixture was used to transform *E. coli* KRX competent cells and positive colonies were confirmed by PCR and DNA sequence analyses.

*H^5^* was cloned as previously described^22^. SNAP-*tag*^®^ and *H^5^* proteins were expressed in *E. coli* ABLE C cells, grown at 37 °C in Luria–Bertani (LB) medium supplemented with 50 mg/l kanamycin and 100 mg/l ampicillin. The protein expression was induced with 1 mM isopropyl-thio-β-D-galactoside (IPTG) at an absorbance value of 0.5–0.6 A_600_ nm. After overnight growth, cells were collected and resuspended 1:3 (w/v) in purification buffer (50 mM phosphate, 300 mM NaCl; pH 8.0) supplemented with 1% Triton X-100 and stored overnight at −20 °C. Subsequently, the biomass was treated in ice with lysozyme and DNAse for 60 min and then sonicated as described (Perugino et al., 2012). After centrifugation of 30 min at 60,000 × *g*, the cell extract was recovered and applied to a Protino Ni–NTA Column 1 ml (Macherey–Nagel) for His_6_-tag affinity chromatography. The eluted fractions containing the protein were collected and dialysed against phosphate-buffered saline (PBS 1×, 20 mM phosphate buffer, NaCl 150 mM, pH 7.3). Pooled fractions were concentrated and protein purification was confirmed by SDS-PAGE analysis. Aliquots were finally stored at −20 °C.

### 2.6 AGTs’ substrate assay by competitive inhibition method

Competitive inhibition assay was performed as described^23^^,^^38^. Briefly, by using a fixed concentration of the fluorescent **SVG** (5 µM) and enzymes (5 µM), an increasing amount of guanine-derivatives (0–2 mM) was added to the mixtures. The reactions were incubated for 30 min at 25 °C and 50 °C for SNAP-*tag*^®^ and *H^5^* respectively, and loaded on SDS-PAGE. Subsequently, fluorescent bands were measured by *gel-imaging* on a VersaDoc 4000™ system (Bio-Rad), by applying a blue LED/530 bandpass filter. Obtained data were finally plotted by Equation (1),

(1) $$\text{RF}=\frac{100\%}{1+{\left(\frac{\left[I\right]}{{\text{IC}}_{50}}\right)}^{\left[S\right]}}$$

where RF is the obtained Relative Fluorescence, [*I*] and [*S*] are the concentration of the inhibitor and the substrate, respectively, and finally the IC_50_ is the concentration needed to reduce by 50% the fluorescence intensity of the protein band.

We evaluated the activity of SNAP-*tag*^®^ and *H^5^* enzymes on **BGN3** and **BGSN3** by the afore-mentioned IC_50_ method (Figure S5(A,B)) and other *O^6^*-guanine-derivatives (Table 1).

**Table 1.** Substrate specificity of SNAP-*tag*^®^ and *H^5^* by competitive inhibition method (IC_50_) by using **SVG** as substrate, and second order rate constant of the enzymatic reaction of these *protein-tags* only on the **BGSN3** substrate.

Structure	Name	SNAP-*tag*^®^		*Ss*OGT-*H^5^*		Note
		IC_50_ (µM)	*k*^a^ (s^−1^ M^−1^)	IC_50_ (µM)	*k* (s^−1^ M^−1^)	
	SVG	–	2.8 × 10^4 b^	–	1.6 × 10^4^	[14,24]
	BG	36.8 ± 5.6	–	10.1 ± 1.0	–	This work
	SCB	2.1 ± 0.5	–	4.4 ± 0.8	–	This work
	BGN3	15.6 ± 0.3	–	23.5 ± 1.0	–	This work
	BG430	ND^c^	–	ND	–	This work
	BGPA	86.0 ± 6.7	–	14.3 ± 1.9	–	This work
	MGPA^d^	–	–	268.9 ± 19.1^e^	–	This work
	BGSN3	17.8 ± 1.1	4.64 ± 1.04 × 10^5^	10.0 ± 0.7	1.40 ± 0.47 × 10^4^	This work

For each compound, the guanine moiety is drawn *in black* and the chemical group conjugated to the benzyl ring *in blue*. The fluorescein moiety of the **SVG** is *in green*, whereas **SCB** differs from the other derivatives by the presence of a benzylic ring (*in red*). Azide group is conventionally coloured *in magenta*.

^a^Reaction rates at 25 °C; ^b^this value was obtained by using a BG-fluorescein substrate (BG-FL) very similar to SVG; ^c^not determined; ^d^this molecule is a *O^6^*-methyl-guanine derivative; ^e^competitive assay for *H^5^* was performed at 65 °C.

### 2.7. In vitro Huisgen Cu(I)-catalysed cycloaddition reaction

The Huisgen chemical reaction was evaluated on SNAP-*tag*^®^ and *H^5^* previously incubated with **BGN3** and **BGSN3**. An opportune amount of purified proteins was incubated within an equimolar ratio of these substrates for 60–120 min at 25 °C and 37 °C respectively, to ensure the complete enzymatic labelling reaction. Later, we performed the subsequent cycloaddition using 5 µM of an alkyne-derivative of the fluorescein (**BDP FL alkyne**), in the presence of copper (1 mM), TCEP (1 mM), TBTA (0.1 mM) and, where indicated, of SDS (0.05%). Finally, mixtures were loaded on SDS-PAGE and analysed as described in Section 4 (Figure S5(C,D)).

### 2.8. Molecular modelling

All molecular modelling studies were performed on a Tesla workstation equipped with two Intel Xeon X5650 2.67 GHz processors and Ubuntu 14.04 (http://www.ubuntu.com). The protein structures and 3D chemical structures were generated in PyMOL (The PyMOL Molecular Graphics System, version 2.2.3, Schrödinger LLC, 2019).

### 2.9. Molecular dynamics (MD) simulation

The MD simulations were carried out using the Desmond simulation package of Schrödinger LLC (Schrödinger Release 2019–1: Desmond Molecular Dynamics System; D. E. Shaw Research: New York, NY, 2019; Maestro-Desmond Interoperability Tools, Schrödinger, New York, NY, 2019). The X-ray structure of the *H^5^* covalently bound to **SVG** was used in this study, entry code 6GA0^39^, water molecules were removed, and all hydrogen atoms and charges were added. The NPT ensemble with the temperature of 300 K and a pressure 1 bar was applied in all runs. The simulation length was 100 ns with relaxation time 1 ps. The OPLS_2005 force field parameters were used in all simulations^40^. The long-range electrostatic interactions were calculated using the particle mesh Ewald method^41^. The cut-off radius in Coulomb interactions was 9.0 Å. The water molecules were explicitly described using the simple point charge model^42^. The Martyna–Tuckerman–Klein chain coupling scheme^43^ with a coupling constant of 2.0 ps was used for the pressure control and the Nosé–Hoover chain coupling scheme^44^ for the temperature control. Non-bonded forces were calculated using an r-RESPA integrator where the short-range forces were updated every step and the long-range forces were updated every three steps. The trajectory sampling was done at an interval of 1.0 ps. The behaviour and interactions between the ligands and protein were analysed using the Simulation Interaction Diagram tool implemented in the Desmond MD package. The stability of MD simulations was monitored by looking at the RMSD of the ligand and protein atom positions in time.

### 2.10. Determination of the rate constants of the chemo-enzymatic labelling approach

Rate constants of the enzymatic reactions with the only **BGSN3** were determined by the method of Gautier et al.^14^. In this case, purified proteins (5 µM) were incubated with the substrate (5 µM) in PBS 1× buffer at 25 °C. Aliquots were taken at different times, the reactions were immediately stopped in Leammli Buffer 1× in addition with 10 µM of **Cy5 DBCO** fluorophore and placing tubes on ice.

Rate constants for the chemical reaction needed for the preliminary achievement of the *clickable*-SNAP and *clickable*-*H^5^* with **BGSN3**, which was obtained by the afore-described protocol, in order to get the complete labelling. Then, to each aliquot of 5 µM of *clickable* proteins, 20 µM of **DBCO-PEG_4_-Fluor 545** fluorophore was added. At different times, an excess of sodium azide (NaN_3_, 300 mM) was immediately added to each aliquot and then placing tubes on ice, in order to stop the click reaction between the azide group on the **BGSN3** and the **DBCO-PEG_4_-Fluor 545** molecule.

**Figure 2.**
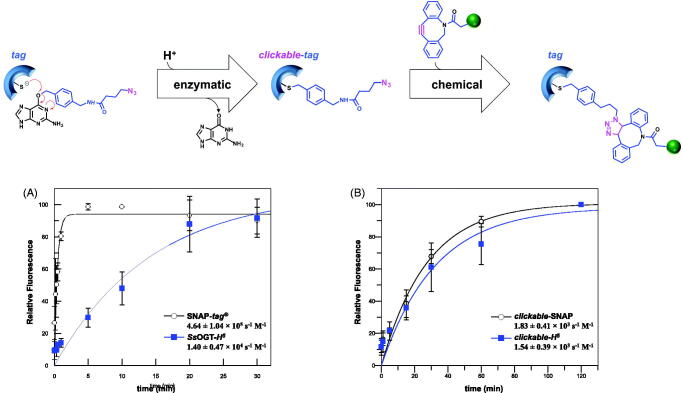
Reaction rates of the chemo-enzymatic approach. Pseudo-first-order reaction of *protein*-*tags* for (A) the enzymatic reaction with **BGSN3** (see *k* values also in Table 1), and of *clickable*-*tags* for (B) Huisgen reaction with **DBCO-PEG_4_-Fluor 545** (see values in the main text). Values given are an average of three independent measurements. The reaction scheme was an exemplification of Figure 1(B) in the main text. Data are represented as mean ± SEM.

Finally, for both the experiments, all aliquots were boiled in an SDS buffer for 5 min, and immediately loaded on a SDS-PAGE, for the *gel-imaging* and *coomassie staining* analyses, as previously described. Data were fitted to a pseudo-first-order reaction model using the GraFit 5.0 software package (Erithacus Software Ltd.). Second-order rate constants *k* (in s^−1^ M^−1^) were then obtained by dividing the pseudo-first-order constant by the concentration of the substrate (Figure 2 and Table 1). Values given are an average of at least three independent measurements.

### 2.11. In vitro Huisgen copper-free cycloaddition reaction with different DBCO-fluorophores

For the copper-free click reaction, aliquots of 5 µM of each *clickable*-protein were incubated for 60 min at room temperature in the dark with 5 µM of fluorescent DBCO-derivative substrates (**BDP FL DBCO**, **Cy5 DBCO**, and **DBCO-PEG_4_-Fluor 545**) in a total volume of 10 µl of PBS 1× buffer (Figure 4 and Figure S6). The reactions were finally stopped in Leammli Buffer 3×, loaded on SDS-PAGE, and analysed as described in Section 4, by applying a blue LED/530 bandpass filter, red LED/695 bandpass filter and green LED/605 bandpass filter as excitation/emission parameters for each DBCO-fluorophores, respectively. The click reaction was also performed on 5 µM of both the enzymes, but in the presence of an *Ec*CFE diluted in PBS 1× buffer.

### 2.12. Procedure for protein immobilisation on bio layer interferometry (BLI), by following the chemo-enzymatic approach

OctetRED96™ (ForteBio, Fremont, CA) was used to immobilise specifically SNAP-*tag*^®^ and *H^5^* with the *chemo-enzymatic approach* (Figure 5(A,B)). Samples and reaction buffers were located in black 96-well plates (OptiPlate-96 Black, Black Opaque 96-well Microplate, PerkinElmer, Billerica, MA) in a maximum reaction volume of 300 µl per well with 800 rpm shaking for each step. For the immobilisation procedure, AR2G sensors were first wetted in 200 µl of pure water for at least 15 min, followed by an equilibration step (3 min) in acetate buffer 0.1 M, pH 5.0. Afterwards, they were activated with 20 mM 1-ethyl-3-(3-dimethyl-amino-propyl) carbodiimide hydrochloride (EDC)/20 mM N-hydroxy-sulfo-succinimide (sulfo-NHS) mixture in acetate buffer (60 min) and covered with 2 mM **propargyl-PEG_3_-amine** bifunctional linker (BroadPharm, San Diego, CA) in Loading step (20 min). To avoid the presence of any free amine groups on the biosensors, a Blocking step with Ethanolamine 1 M (30 min) was performed. Subsequently, a Washing step (15 min) with water and an Equilibration step in click-reaction buffer (15 min) are followed.

During the afore-described procedure, proteins were labelled with **BGSN3**. Finally, the immobilisation step for each sample via Huisgen reaction was carried out at 30 °C for 80 min, followed by a Washing step (20 min), in order to remove all the unbound molecules. This procedure was the same in the presence of the *Ec*CFE. All measurements were performed in triplicates.

### 2.13. Permeability of eukaryotic and prokaryotic cells to BGSN3

HEK293T cells were maintained at 37 °C with 5% CO_2_ in Dulbecco’s Modified Essential Medium (Invitrogen, Carlsbad, CA) supplemented with 10% Foetal Bovine Serum (FBS) (Invitrogen) and 100 U/ml Penicillin/Streptomycin (Roche, Switzerland). HEK293T cells were transfected with SNAP-*tag*^®^ plasmid by using Lipofectamine 2000 (Invitrogen) following manufacturer’s protocol. The treatment with **BGSN3** were performed, at the concentration and time indicated for each experiment. Twenty-four hours after transfection, we treated cells with **BGSN3** for 2 h at different concentrations ranging (from 1 to 25 µM), directly dissolving the compound in complete culture medium. Then cells were harvested, washed with PBS 1× buffer and lysed with 50 mM Tris-HCl pH 7.4, 150 mM NaCl, 0.5 mM EDTA, 0.1% Triton X-100 supplemented with complete protease (Roche, Switzerland) and phosphatase (SERVA Electrophoresis, Germany) inhibitors. Afterwards, transfected cells were treated with a fixed concentration of **BGSN3** (10 µM) at different time points (from 30 to 120 min). Again, HEK293T cells were washed and lysed as described before. To confirm the reaction with **BGSN3**, the same amount of protein extract (0.91 µg/µL for each sample) was incubated for 30 min at 25 °C with **SVG**. Subsequently, proteins were loaded on SDS-PAGE and analysed by *gel-imaging* on a VersaDoc 4000™ system (Bio-Rad), by applying a blue LED/530 bandpass filter (Figure 6).

For flow cytometry analysis, HeLa cells were seeded in 24-well plates and transfected with SNAP-*tag*^®^ plasmid by using Lipofectamine 2000 (Invitrogen, USA) following manufacturer’s protocol. Twenty-four hours after the transfection, cells were treated with 25 µM **BGSN3** for 1 h, and the excess of the substrate was washed out by 2 × 15 min, followed by 1 × 30 min washes. Cells where then treated with 2.5 µM **BDP FL DBCO** for 30 min and unbound fluorophore was removed by following the same procedure performed for the **BGSN3**. All treatments and washes were performed at 37 °C in a complete culture medium. Lastly, cells were harvested by trypsinization, and fluorescence was measured using FACS CANTO II instrument. The analysis was performed on live singlet cells using FlowJo software (Figure S7(A)).

*E. coli* ABLE C strain was transformed with SNAP-*tag*^®^ plasmid and protein expressed as previously described. After overnight growth, samples of 2 ml were treated with 100 µM of **BGSN3** for 2 h at 25 °C and then collected by centrifugation at 2000 × *g*. Cell pellets of 0.05 g were resuspended 1:3 (w/v) in PBS 1× supplemented with 1% Triton X-100 and subjected to cell lysis, by applying 5 cycles of freeze-thawing. After a centrifugation at 13,000 × *g*, the supernatants containing the protein extract were incubated 30 min at 25 °C with **SVG,** and proteins were loaded on SDS-PAGE. Finally, fluorescent bands were analysed by *gel-imaging* techniques (Figure S7(B)).

## 3. Result and discussion

### 3.1. Substrate specificity of AGTs on BG-based substrates

Following the irreversible reaction shown in Figure 1, we evaluated the activity of two enzymes in our possession on several *O^6^*-guanine-derivatives (Table 1). Because most of them are non-fluorescent compounds, we performed an AGTs’ competitive inhibition assay by using the fluorescein-derivative SNAP-Vista^®^ Green as substrate (**SVG**), as previously described^22–24^^,^^45^. Briefly, the reaction of an AGT with **SVG** led to a fluoresceinated protein, which can be visualised as a fluorescent band in *gel-imaging* analysis after SDS-PAGE. The presence of increasing amounts of a non-fluorescent competitor in the reaction causes a decrease of the fluorescent signals, which can be measured and plotted for the IC_50_ values determination^23^^,^^46^. As shown in Table 1, SNAP-*tag*^®^ and *H^5^* displayed different behaviours versus these competitors, without any rationale for the dimension and/or polarity of the conjugated chemical groups. While SNAP-Cell^®^ 430 (**SC430**) completely lost the competition with **SVG**, both the enzymes are extremely active on the SNAP Cell^®^ Block (**SCB**), displaying the lowest IC_50_ value measured. This result was expected, because **SCB** has a structure very similar to the Lomeguatrib, one of the most efficient inhibitors of the hMGMT protein, employed in the cancer treatment in combination with alkylating agents-based chemotherapeutics^47^.

In general, all commercially available products used (**SVG**, **SCB**, **BG430**, and **BG-PEG-NH2**, **BGPA**) are good substrates for the SNAP-*tag*^®^ and *H^5^* enzymes, completing their labelling reaction in few hours (data not shown). However, based on our results, the choice of the chemical group to be conjugated to the *O^6^*-guanine for zcustomized substrates may present risks, with consequent decreases in the reaction rate for these *protein-tags*. This was the case of methyl-guanine-PEG-NH2 (**MGPA**), which is an *O^6^*-methyl-guanine derivative, used for the immobilisation of SNAP-*tag*^®^ on nanoparticles^48^. The latter is not a preferred substrate, probably because of the absence of the benzyl ring, which leads to complete labelling of the SNAP-*tag*^®^ and *H^5^* after over-night incubation at 4 °C^48^ and 65 °C (data not shown), respectively.

### 3.2. In vitro enzymatic reaction of engineered AGTs with BG-azide substrates

Recent studies were focussed on the synthesis of alternative “BG-building blocks,” which offer the opportunity to produce SNAP-substrates by following easier and faster protocols: an alkyne substituted *O^6^*-BG was employed in the synthesis of compounds by the Huisgen cycloaddition with azide-based fluorescent probes^49^ or, inversely, by using the *O^6^*-BG-N_3_ (**BGN3**, Figure S1) for the conjugation with alkyne-based chemical groups^34^. We evaluated the enzymatic reaction of the *H^5^* and the SNAP-*tag*^®^ *directly* on **BGN3** and a synthesised BG-derivative containing a benzyl ring opportunely spaced from the azide group (**BGSN3**, Figure S4): after the reaction, no fluorescent signal was obtained on SDS-PAGE *gel-imaging* upon the addition of **SVG** (Figure S5(A,B)). This indicates that the catalytic cysteine was completely blocked by the benzyl-azide moiety, impeding the access of the fluorescent substrate to the active site. Compared to the classical BG-derivatives, these *protein-tags* showed a reasonable activity on both these BG-azides, as resulted by the calculated IC_50_ (Table 1 and Figure S5(A,B)).

After the enzymatic reaction of *H^5^* with **BGN3** and **BGSN3**, we performed the subsequent cycloaddition using an alkyne-derivative of the fluorescein (**BDP FL alkyne**): however, the chemical reaction was less efficient using the former substrate (Figure S5(C), lane 2). In this case, the complete fluorescein labelling of the protein was achieved only in the presence of a small amount of SDS during the cycloaddition step (lane 3), suggesting that the protein is still folded after the enzymatic reaction and the azide is hidden in the active site core. The addition of the denaturant could have slightly opened the protein structure, favouring a better exposure of the azide group to the solvent, and allowing the click reaction to occur.

On the contrary, using **BGSN3** as substrate, the labelling of both the enzymes was comparable to the classical reaction with **SVG** without any denaturing agent, likely the longer spacer of **BGSN3** could sufficiently move away from the azide group from the protein surface for the Huisgen reaction (Figure S5(D), lanes 2 and 4). From now on, experiments were only performed by using the longer BG-azide. We first calculated the rate of the enzymatic reaction, demonstrating that both *protein-tags* show a high catalytic activity comparable to the commercial BG-derivatives currently used (Figure 2(A) and Table 1), also indicating that the complete protein labelling in less of an hour can be performed^13^^,^^14^^,^^24^.

### 3.3. Molecular modelling on the H^5^ with BG-azides

**Figure 3.**
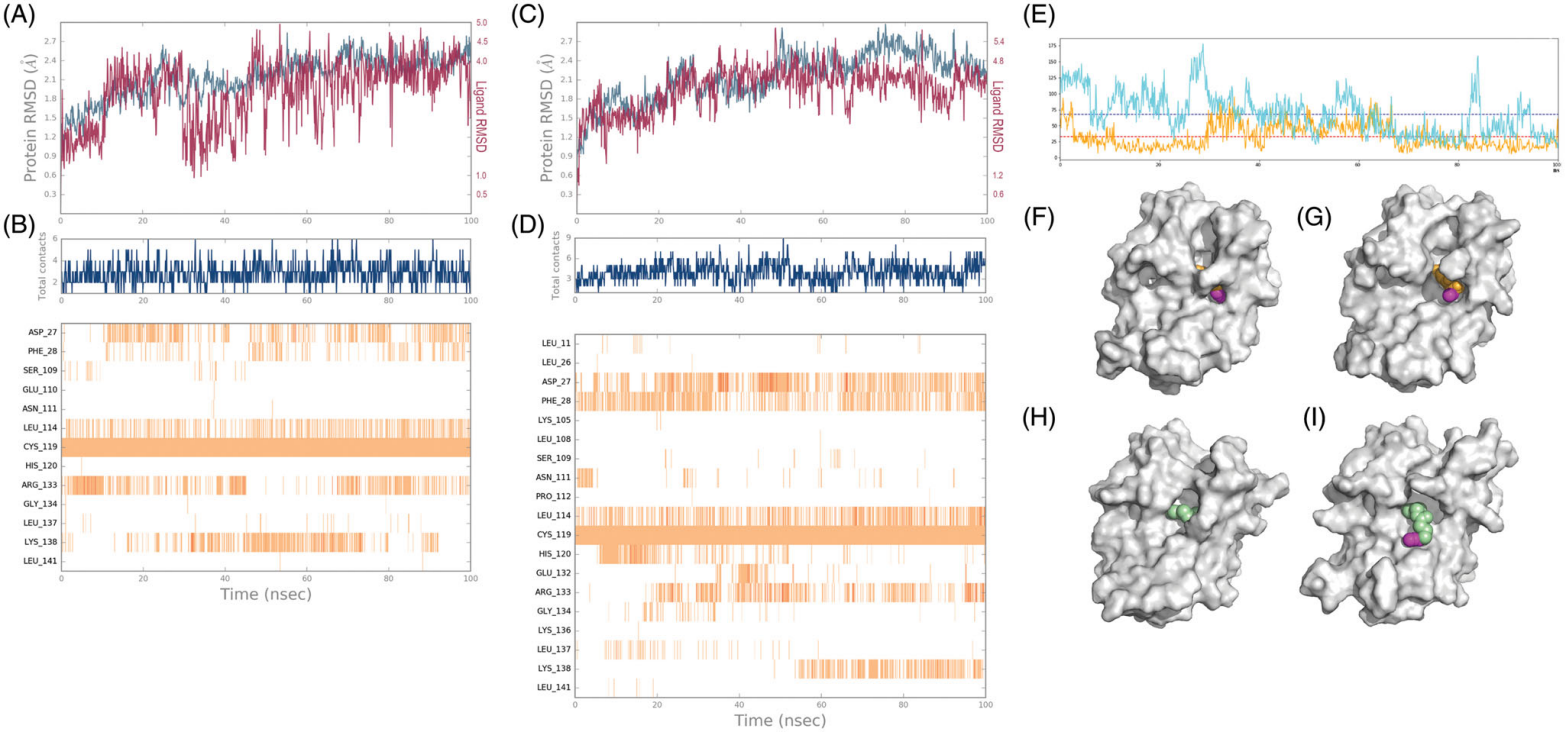
Molecular modelling on *H^5^* with BG-azides. (A) RMSD of the atomic positions for the compound **BGN3** (Lig fit Prot, *in red*) and the protein *H^5^* (Cα positions, *in blue*) of the 100 ns molecular dynamics simulations using Desmond package. (B) A timeline representation of the interactions and contacts (H-bonds, Hydrophobic, Ionic, Water bridges). (C) RMSD of the atomic positions for the compound **BGSN3** (Lig fit Prot, *in red*) and the protein *H^5^* (Cα positions, *in blue*) of the 100 ns molecular dynamics simulations using Desmond package. (D) A timeline representation of the interactions and contacts (H-bonds, Hydrophobic, Ionic, Water bridges). (E) Solvent Accessible Surface Area (SASA) of **BGN3**/*H^5^* (*in orange*) and **BGSN3**/*H^5^* (*in cyan*) complexes over the MD simulation time (mean values are depicted as dot lines). Frames of *H^5^*-probe complexes with lower (F, H) and higher (G, I) SASA value for **BGN3** (F, G) and **BGSN3** (H, I), respectively.

**BGN3** and **BGSN3** differ in length since the chemical spacer between the benzyl ring and the active azide makes the latter potentially more prone to the labelling reaction. It could be assumed that this aspect alone influences the availability of the azide moiety to react. However, proteins are not a static system, the amino acids side-chain movements could mask the azide and prevent the “click” chemistry reaction. The covalent complexes of these compounds with *H^5^* were analysed with Molecular Dynamics (MD) simulations using the Desmond package (see Experimental Section). The complexes were simulated for 100 ns at 300 K using a standard protocol. The protein structure has been stabilised, as shown in the RMSDs for both the IDO1 Cα and the ligand (Figure 3(A,C)). The MD results were analysed in terms of Solvent Accessible Surface Area (SASA) of the compounds: more time the compounds are exposed to the solvent, the higher is the possibility to react^50^. In Figure 3 is reported the fluctuation of the SASA values over the simulation time together with the structure model of the *H^5^* protein in complex with **BGN3** and **BSGN3**, respectively. The former is less exposed to the solvent with a SASA value of 32.967 ± 18.573 Å^2^ compared to **BGSN3**, which shows a higher SASA value 68.302 ± 32.455 Å^2^. This simulation confirmed our biochemical data, proposing the BG-derivative with the spacer as a better substrate for our *chemo-enzymatic approach*.

### 3.4. Specificity and versatility of the chemo-enzymatic reaction

The *O^6^*-BG-based **BGSN3** is a good substrate for the two *protein-tags* used (Table 1 and Figure 2(A)) and offering the advantage to sufficiently expose the azide group for the Huisgen reaction. This was the starting point to examine: (i) the labelling efficiency of the *clickable*-SNAP and *clickable*-*H^5^* by using different DBCO-based fluorophores; (ii) the specificity of the “click” reaction.

**Figure 4.**
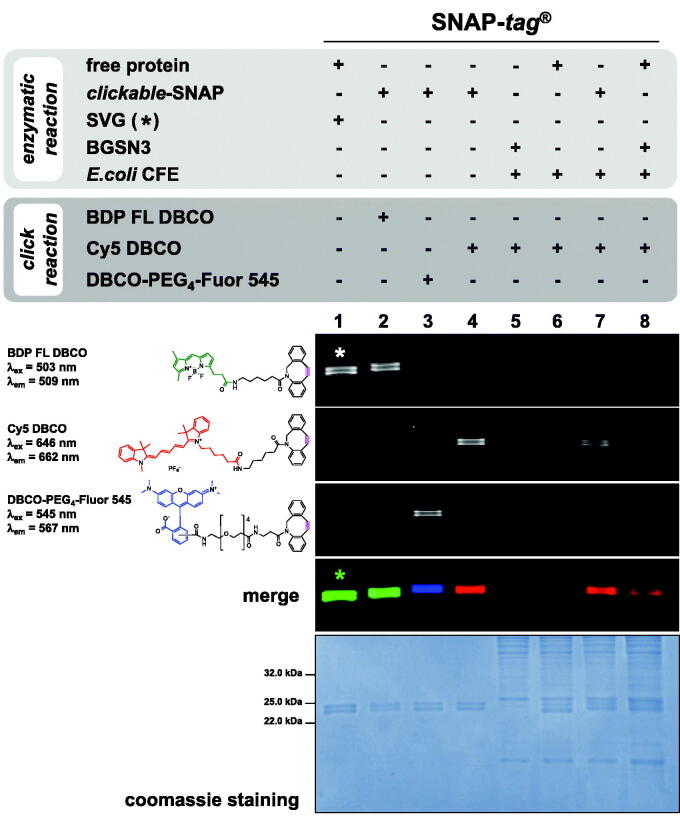
Specificity of the Huisgen reaction. *Gel-imaging* analysis of SNAP-*tag*^®^ labelling by a *chemo-enzymatic approach* with **BGSN3** and three different DBCO-derivative fluorophores. Protein (5 µM) was incubated with 5 µM of the azide-based BG for 60 min at 25 °C; then, an equimolar amount of DBCO-based substrate was added for the chemical click reaction, keeping the same time and temperature conditions. As control, SNAP-*tag*^®^ was incubated only with **SVG** (lane 1, signal marked with an asterisk).

Upon the reaction with **BGSN3**, all cycloaddition reactions with three different DBCO-based fluorophores were complete in ca. 30–45 min in PBS 1× buffer (Figure 4, lanes 2–4), with a protein-labelling as efficient as the enzymatic reaction using the sole **SVG** (lane 1). We quantitatively evaluated the rate (*k*) of the click reaction by using the **DBCO-PEG4-Fluor 545** fluorophore: as expected, both the *clickable-tags* were labelled with the same efficiency (1.83 ± 0.41 × 10^3^ s^−1^ M^−1^ for SNAP-*tag*^®^; 1.54 ± 0.39 × 10^3^ s^−1^ M^−1^ for *H^5^*), demonstrating that the chemical reaction is sufficiently fast and independent from the *tags* (Figure 2(B)).

Concerning the specificity, we added a crude protein extract from *Escherichia coli* ABLE C (*Ec*CFE), without any AGT activity at the *gel-imaging* analysis (Figure 4, lane 5). In this context, the only presence of the free *protein-tag* and the DBCO-fluorophore also did not result in any fluorescent signal (lanes 6), whereas the previously purified *clickable*-SNAP (lane 7), as well as its free form in the presence of **BGSN3** (lane 8), was specifically able to complete the chemo-enzymatic reaction, giving an evident fluorescent signal. The high specificity of our approach was also confirmed by using the *H^5^* enzyme, which displays a better labelling reaction than the mesophilic SNAP-*tag*^®^ (Figure S6). Probably, something in the extract might impede SNAP-*tag*^®^ activity. These results clearly demonstrated the high efficiency of our *chemo-enzymatic approach* for the labelling of both the *protein-tags* used.

### 3.5. Application to the bio layer interferometry

**Figure 5.**
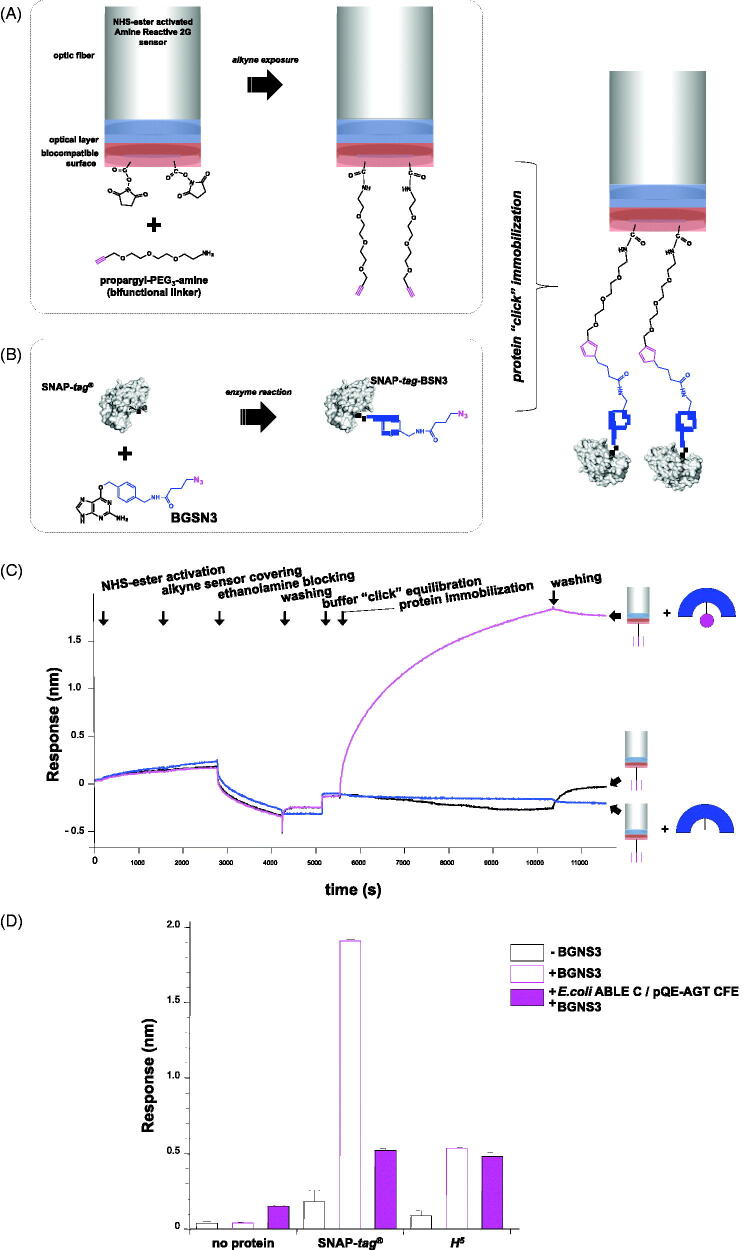
Covalent immobilisation of clickable-tags on the BLI sensor. (A) Covering of the BLI sensor with a bi-functional linker, exposing alkyne groups for the Huisgen cycloaddition reaction; (B) reaction of the SNAP-*tag*^®^ with **BGSN3**; (C) chemo-enzymatic SNAP-*tag*^®^ immobilisation on BLI. The alkyne-covered sensor (*silver cylinder*) was immersed in wells containing the buffer (*in black*), the free SNAP-*tag*^®^ (*in blue*) and the *clickable*-SNAP (*in magenta*); (D) column chart relative to the BLI immobilisation of purified *protein-tags* alone (*black-bordered bars*) or in the presence of **BGSN3** (*magenta-bordered bars*). Filled magenta bars represent the BLI immobilisation using the *Ec*CFE upon heterologous expression of *protein-tags*. Standard deviations were obtained from three independent experiments. Data are represented as mean ± SEM.

The possibility to apply the SNAP-*tag*^®^ technology to the Surface Plasmon Resonance (SPR) for the covalent immobilisation of a protein of interest was first explored by the group of Kai Johnsson^37^, followed by other groups with the same substrate^51^ or a biotin BG-derivative^52^. Their approaches, again, required preliminarily the synthesis and the purification of a compatible substrate to cover the sensor chip surface. We used, instead, the **BGSN3** substrate for the immobilisation of the SNAP-*tag*^®^ directly on an alkyne-derived sensor chip of the bio layer interferometry (BLI) equipment, as shown in Figure 5. This technique is more advantageous with respect to the SPR because: (i) it needs a smaller amount of sample, making it more compatible to higher throughput (the capacity of running up to 96 samples in a parallel); (ii) the possibility to reuse samples, and (iii) of the total independency from any microfluidic issues.

Given the lack of any available BLI alkyne-derived sensors, we first activated the AR2G type by a bi-functional linker (**propargyl-PEG 3-amine**) in order to expose an alkyne group on the surface (Figure 5(A)). This modified protocol provides first the coating of the sensor tips with alkyne groups (approx. 80 min), during that the reaction between the *protein-tag* and **BGSN3** inside the 96-wells rack takes place (Figure 5(B)). Only the contemporary presence of the *clickable*-SNAP and the alkyne-coated sensor led to a measurable response (Figure 5(C)). After washing procedures, the signal did not significantly drop-down, given the covalent reaction between the protein and the sensor. We successfully achieved results with both the enzymes, although temperature and times of the enzymatic reaction on BLI (30 °C) favoured the SNAP-*tag*^®^ respect to the thermophilic *H*^5^^13^^,^^24^. Furthermore, in *Ec*CFEs where both the enzymes were expressed, a specific and efficient immobilisation on BLI sensor tips occurred (Figure 5(D)), although the SNAP-*tag*^®^ displayed a lower labelling efficiency in the *Ec*CFE, as expected (compare lane 8 in Figure 4 and Figure S6). As for other techniques, this specific surface immobilisation of SNAP-*tag*^®^ gives the opportunity to perform a directly *on-chip purification* of a tagged-POI from a crude lysate. without any purification step, in an indirect manner, which favours a better orientation of the POI for its biological activities.

### 3.6. Permeability of eukaryotic and prokaryotic cells to BG-azides

**Figure 6.**
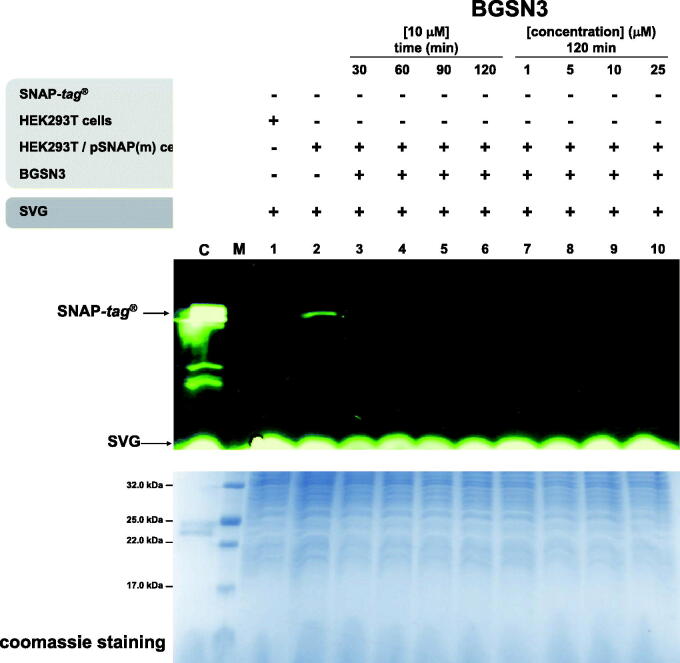
Eukaryotic permeability to BGSN3. SDS-PAGE analysis by *gel-imaging* and *coomassie staining* of HEK293T cell lysates. After **BGSN3** in medium treatment, lysates were incubated with **SVG**.

One of the major applications of the SNAP-*tag*^®^ technology concerns the field of cell biology, where detecting fluorescent-tagged-POIs in living cells represents an important tool to study protein functions and locations^53^. To test our *chemo-enzymatic approach*, we first investigated the permeability of **BGSN3**. Lysates of HEK293T cells pre-treated with **BGNS3** were then incubated with the **SVG** substrate: the absence of any fluorescent signal by *gel-imaging* only in BG-azide treated lysates demonstrated that the internalisation of **BGSN3** was fast (ca. 30 min; Figure 6, lane 3) and at concentrations comparable with commercial cell biology BG-substrates (in the range of <5 µM; Figure 6, lane 8). Preliminary experiments by FACS analysis confirmed that the *in vivo* cycloaddition between **BGSN3** and the **BDP-FL DBCO** occurred (Figure S7(A)). This was also confirmed for *E. coli* bacterial cells (Figure S7(B), lane 2).

## 4. Conclusions and perspectives

We developed an innovative modification of the SNAP-*tag*^®^ technology, in order to overcome times and costs relative to the production and the utilisation of commercial or purified customised BG-derivatives. Although they are compatible in terms of catalytic activity as for the SNAP-*tag*^®^, as well as for the others AGTs^22–24^^,^^37^^,^^46^^,^^54^ the risk of lowering the catalytic activity of these *tags* with customised BG-derivatives should not be underestimated (Table 1). We started by the knowledge that: (i) self-labelling *protein-tags* are still folded and enough stability in their benzylated form after the enzymatic reaction^13^^,^^24^; (ii) the Huisgen cycloaddition is extremely versatile, fast and specific. Recently, the latter was used for the entrapment of catalytic activities by azide-based pseudo-substrates in a well-known powerful method, the *in vivo* activity-based protein profiling (ABPP)^55^. For these reasons, a *chemo-enzymatic approach* (Figure 1(B)) with an opportunely selected azide-based BG-substrate (**BGSN3**) was set up: the efficient exposition of the azide outside the protein surface allows the reaction with a huge number of commercially DBCO-based molecules, more than those BG-derivatives, keeping high the specificity in the presence of *in vitro* “perturbing” proteins (like in cell lysates) and the *in vivo* labelling of expressed SNAP-*tag*^®^ in eukaryotic cells. Finally, **BGSN3** proved to be a good substrate for the direct immobilisation of these *tags* on solid surfaces. We demonstrated that splitting the SNAP-*tag*^®^ reaction into two fast steps, as experimentally measured (Figure 2(A,B)), does not affect the overall rate and efficiency of the protein labelling^13^^,^^24^, thus opening new perspectives and widening the applications of this powerful biotechnology.

## Authors’ contributions

Conceptualisation, G.P. and A.Mi.; Methodology, R.Me., G.P.; Investigation, R.Me., D.C. and M.C.; Formal Analysis, C.M., R.Mi. and A.Ma.; Validation, R.Me., R.Ma., A.Mi. and G.P.; Writing – Original Draft, R.Me. and G.P.; Writing – Review & Editing, C.M., F.R. and A.V and G.P.; Funding Acquisition, A.V. and G.P.; Resources, R.Me, R.Ma. and D.C.; Supervision, A.L., A.Mi. and G.P.

## Supplementary Material

Supplemental Material
