# Supplementary material for: The SNAP-tag technology revised: an effective chemo-enzymatic approach by using a universal azide-based substrate
Source: J Enzyme Inhib Med Chem. 2020 Oct 29;36(1):85–97. doi: 10.1080/14756366.2020.1841182 (PMC7599001; doi:10.1080/14756366.2020.1841182)
Supplement: Supplemental Material [file IENZ_A_1841182_SM7385.zip › Merlo et al_JEIMC 2020_SI_revised.pdf]

The SNAP-*tag* technology revised: an effective *chemo-enzymatic approach* by using a universal azide-based substrate

Rosa Merlo,[‡a] Diego Caprioglio,[‡b] Michele Cillo,[‡c] Anna Valenti,[a] Rosanna Mattossovich,[a] Castrese Morrone,[b] Alberto Massarotti,[b,d] Franca Rossi,[b] Riccardo Miggiano,[b,d] Antonio Leonardi,[c] Alberto Minassi,*[b] and Giuseppe Perugino**[a]

[a]Institute of Biosciences and BioResources, National Research Council of Italy, Via Pietro Castellino 111, 80131 Naples, Italy; [b]Department of Pharmaceutical Sciences, University of Piemonte Orientale, Largo Donegani 2/3, 28100 Novara, Italy; [c]Department of Molecular Medicine and Medical Biotechnology, University of Naples "Federico II", Via Pansini 5, 80131 Naples, Italy; [d]IXTAL srl, Via Bovio 6, 28100, Novara, Italy.

SUPPORTING INFORMATION

FIGURE TITLES AND LEGENDS

Figure S1. *BGN3 spectroscopic characterization.*

Figure S2. *Scheme of the synthesis of the BGSN3 substrate.*

Figure S3. *Compound 3 spectroscopic characterization.*

Figure S4. *BGSN3 spectroscopic characterization.*

Figure S5. *Specificity of BG-azides for the chemo-enzymatic approach.* IC_{50} plots of the competitive fluorescent inhibition of SNAP-*tag*® and H^5 by **BGN3** (A) and **BGSN3** (B), using **SVG** as substrate (see IC_{50} values on Table 1). Values obtained from three independent experiments. Gel-imaging SDS-PAGE of the chemo-enzymatic reaction with BG-azides. Proteins were first incubated at appropriate temperatures (see Experimental) in the presence of **BGN3** (C) or **BGSN3** (D). After the enzymatic reaction, the fluorescent **BDP FL alkyne** and all components for the Huisgen cycloaddition were added to the mixture. As control, each protein was incubated only with **SVG**.

Figure S6. *Specificity of the Huisgen reaction*. *Gel-imaging* analysis of H^5 labelling by a *chemo-enzymatic approach* with **BGSN3** and three different DBCO-derivative fluorophores. Protein (5 µM) was incubated with 5 µM of the azide-based BG for 60 min at 37 °C; then, an equimolar amount of DBCO-based substrate was added for the chemical click reaction, keeping the same time and at room temperature. As control, H^5 was incubated only with **SVG** (lane 1, signal marked with an asterisk).

Figure S7. *Eukaryotic and procaryotic cell permeability to BGSN3.* (A) FACS analysis of HeLa cells pre-treated with the **BGSN3** and then incubated with the **BDP FL DBCO fluorophore** (see text for the procedure). (B) SDS-PAGE analysis by gel-imaging and coomassie staining of lysates from *E. coli* ABLE C cells. After **BGSN3** in medium treatment, lysates were incubated with **SVG**.
