# Supplementary figures and images for: The SNAP-tag technology revised: an effective chemo-enzymatic approach by using a universal azide-based substrate
Source: J Enzyme Inhib Med Chem. 2020 Oct 29;36(1):85–97. doi: 10.1080/14756366.2020.1841182 (PMC7599001; doi:10.1080/14756366.2020.1841182)

Figure S1

A

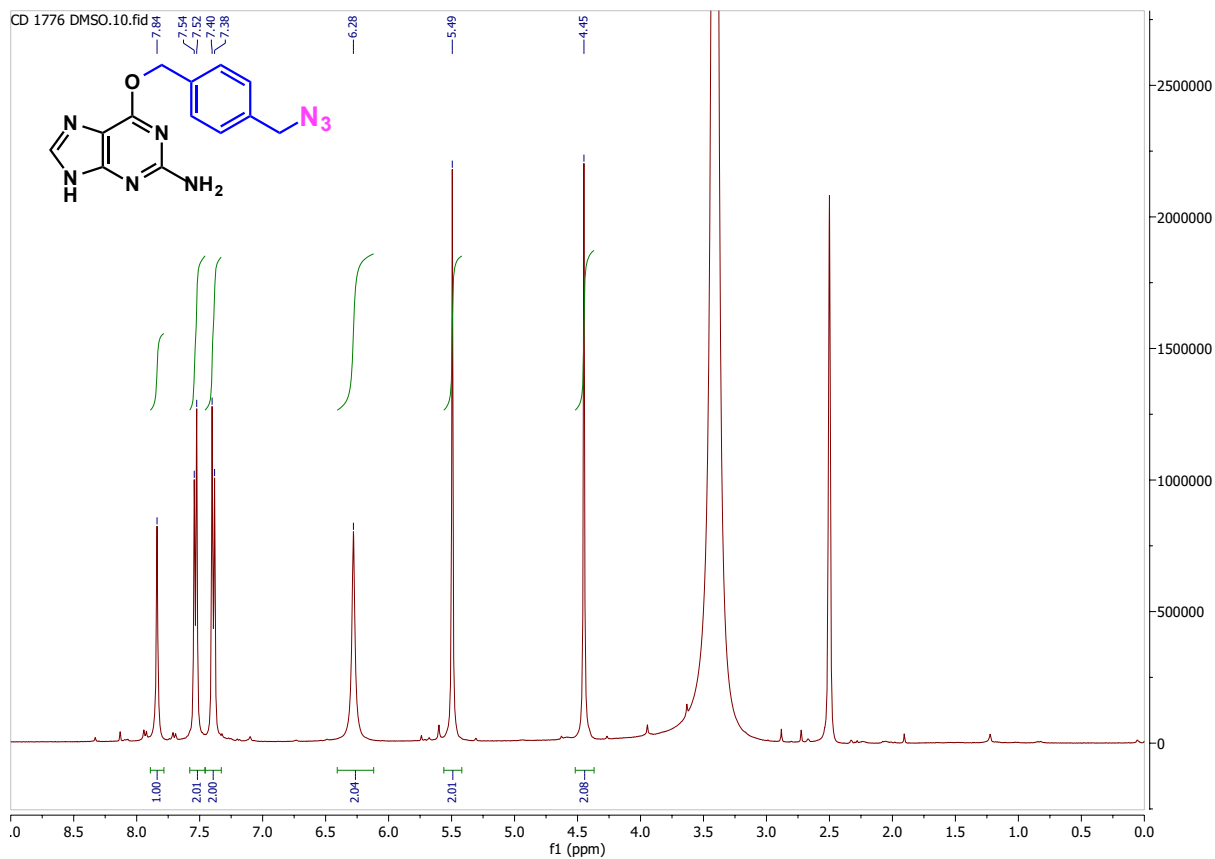

B

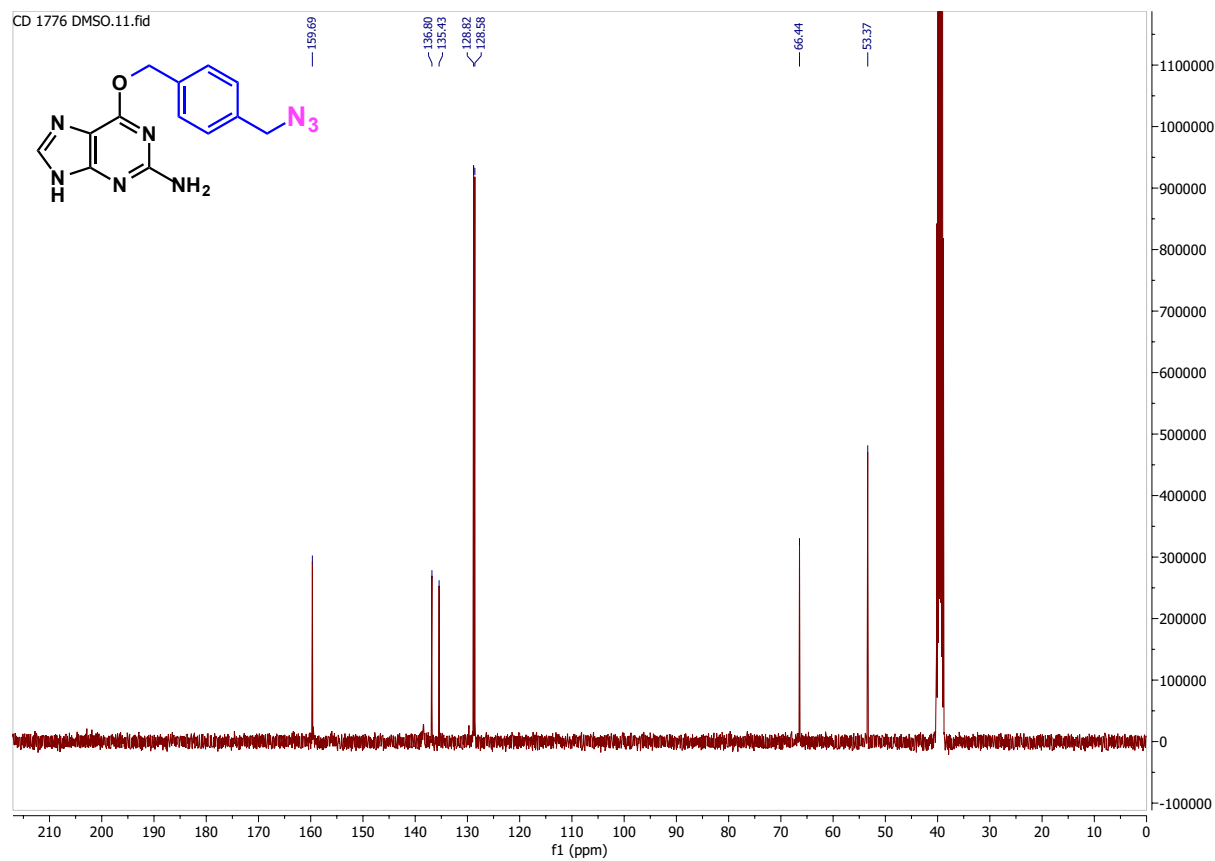

Supplement: Supplemental Material [file IENZ_A_1841182_SM7385.zip › 10 Merlo et al Figure S1.pdf]

Figure S2

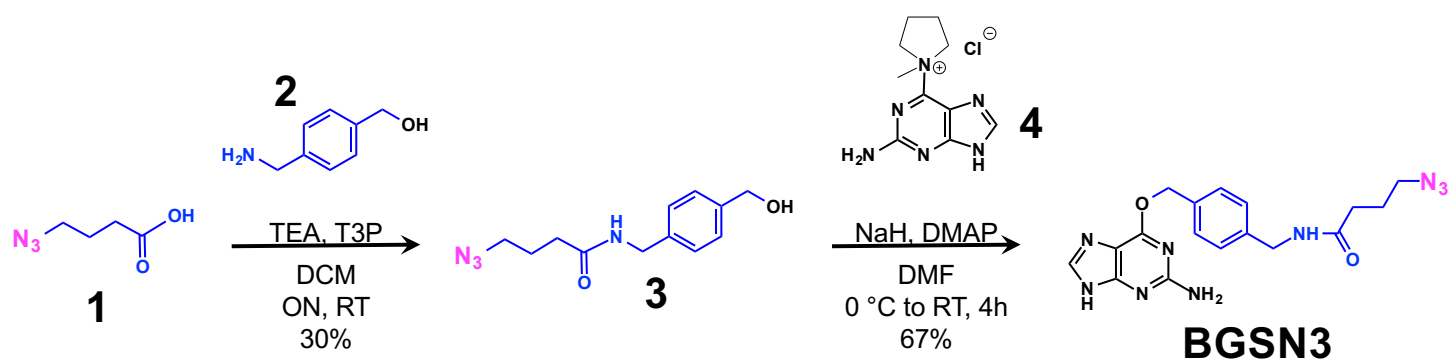

Supplement: Supplemental Material [file IENZ_A_1841182_SM7385.zip › 11 Merlo et al Figure S2.pdf]

Figure S3

A

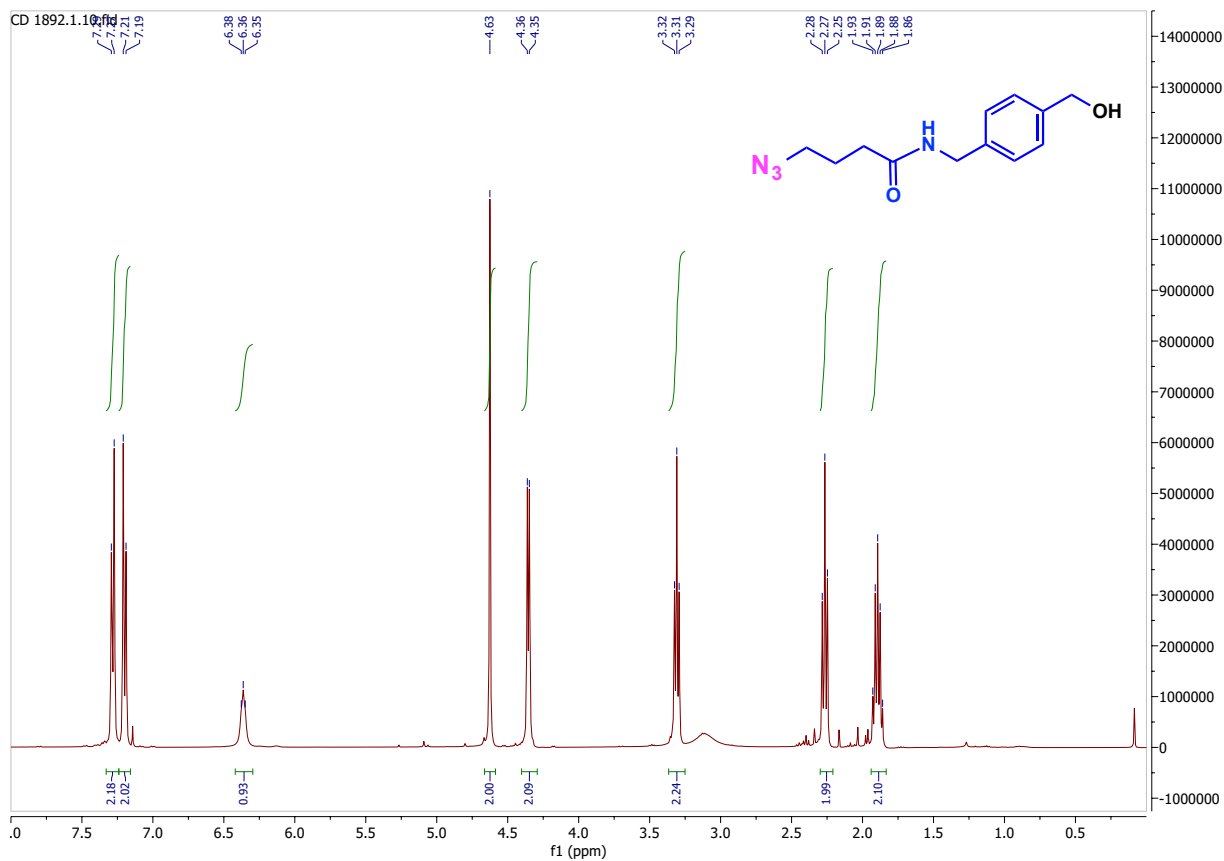

B

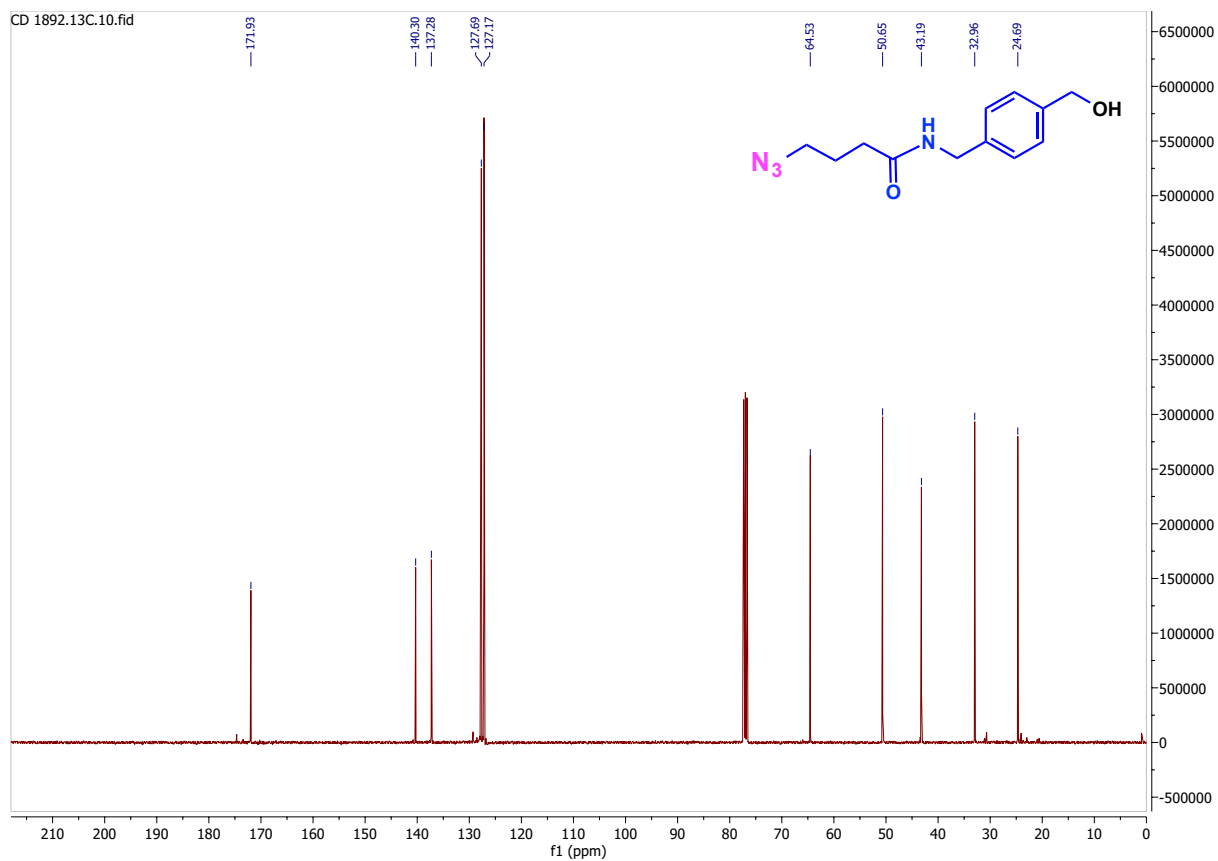

Supplement: Supplemental Material [file IENZ_A_1841182_SM7385.zip › 12 Merlo et al Figure S3.pdf]

Figure S4

A

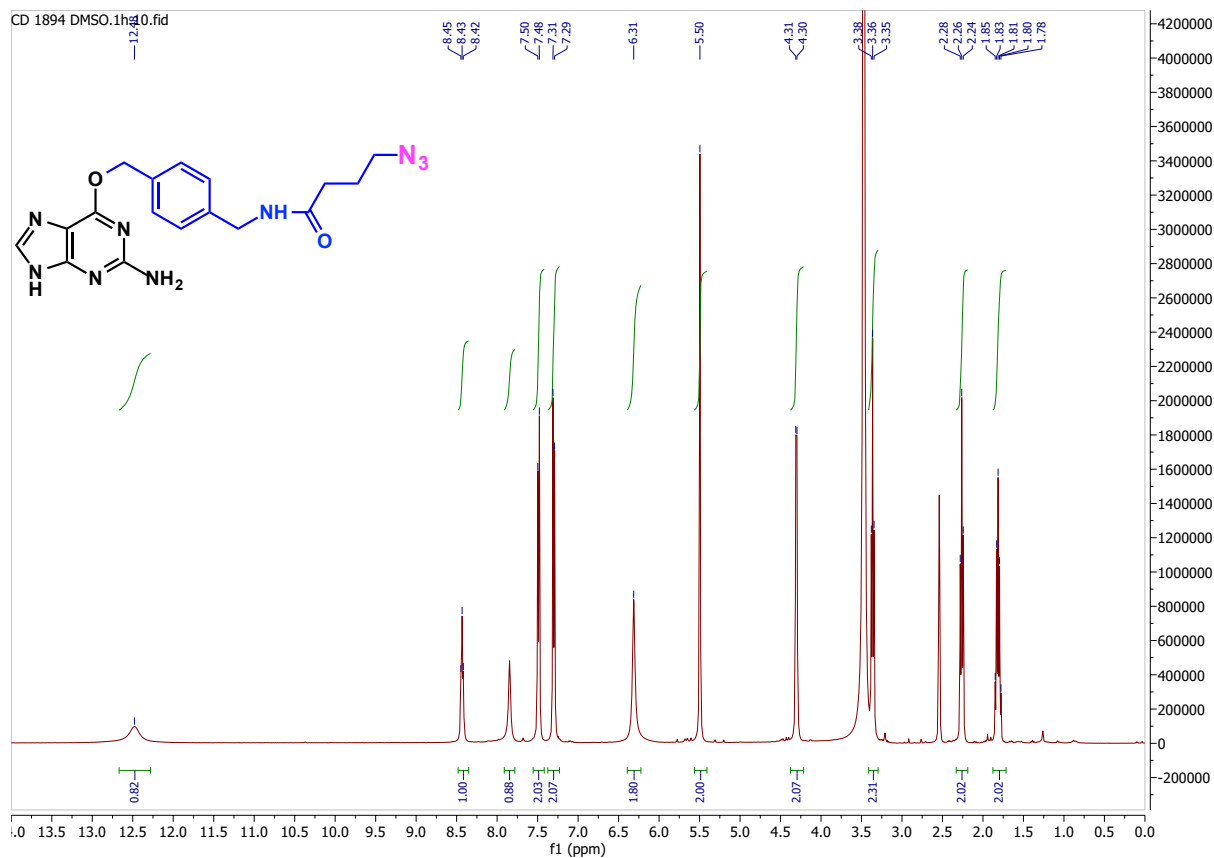

B

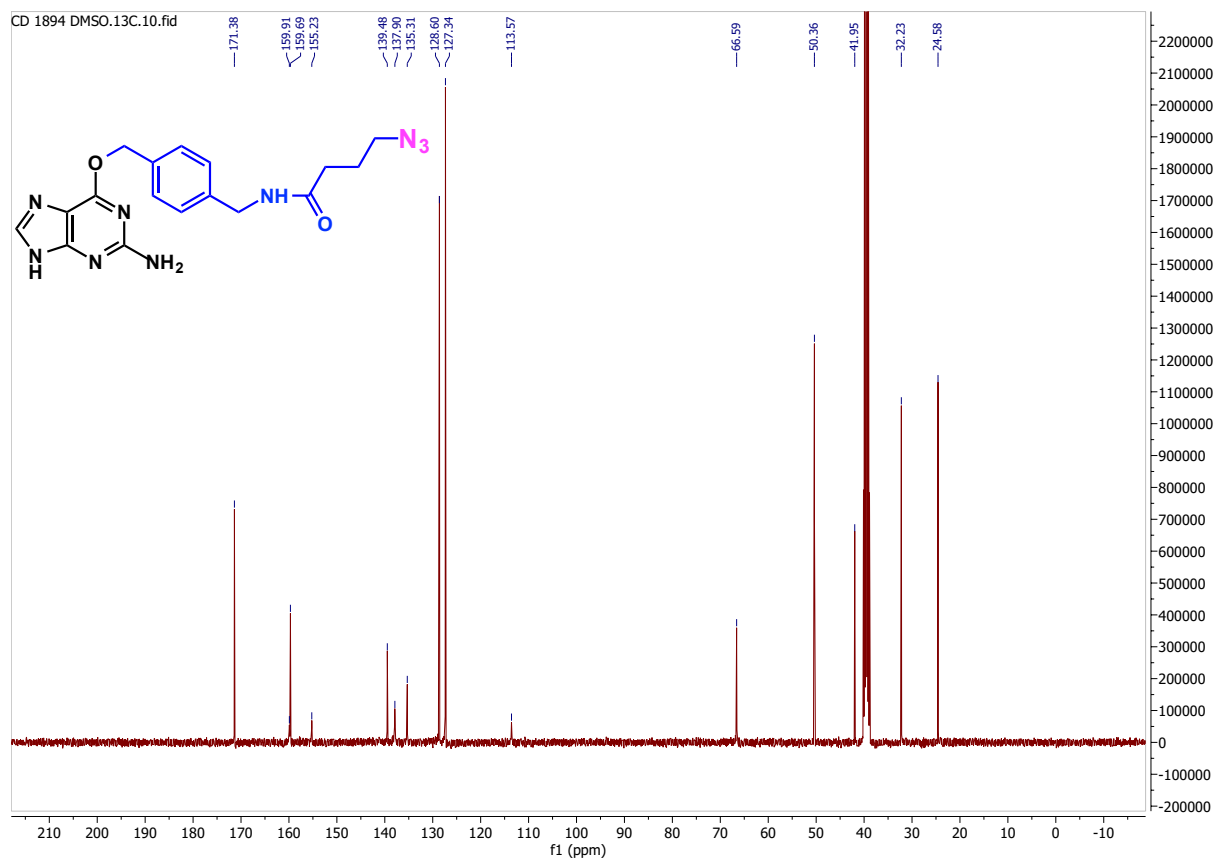

Supplement: Supplemental Material [file IENZ_A_1841182_SM7385.zip › 13 Merlo et al Figure S4.pdf]

# Figure S5

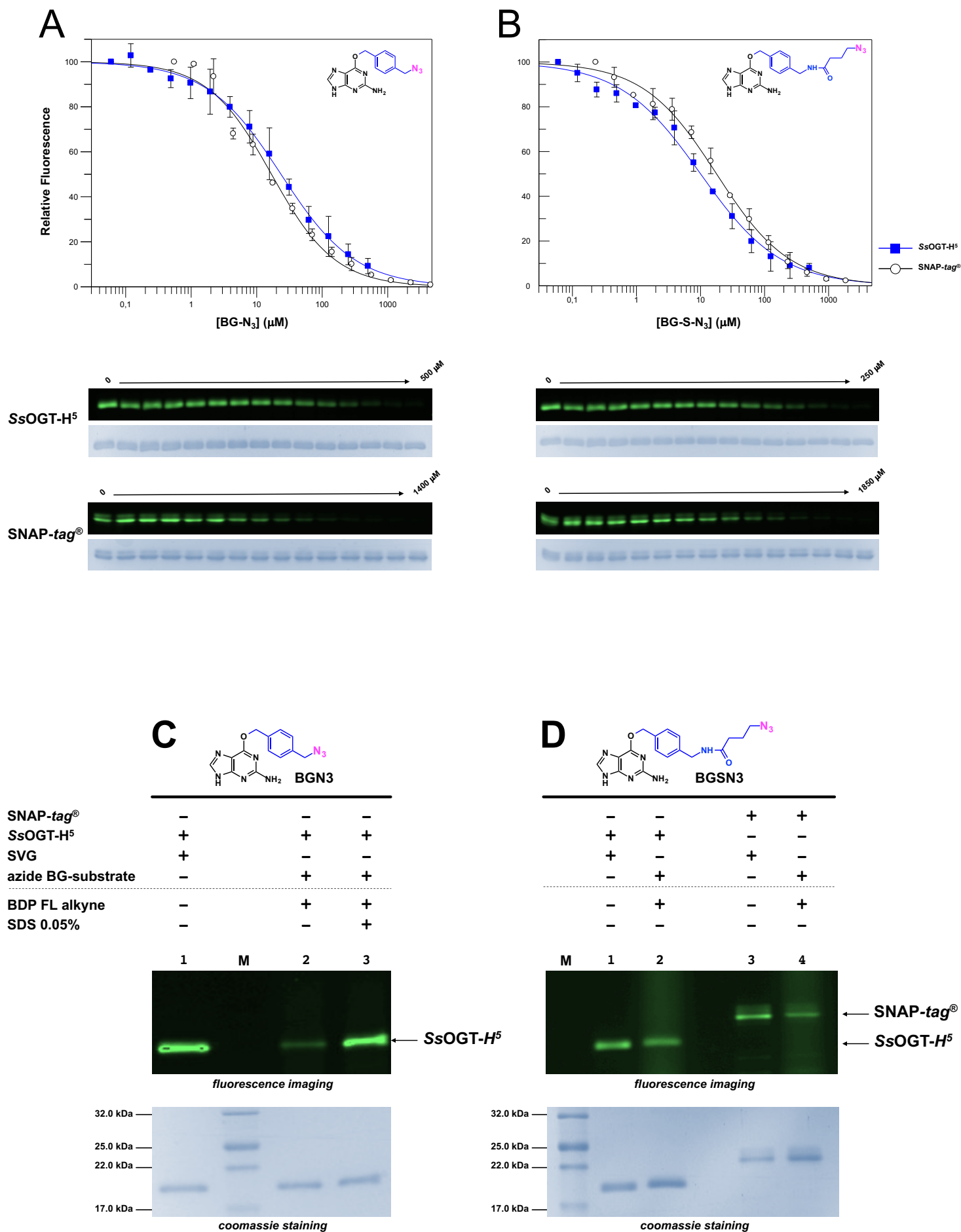

Supplement: Supplemental Material [file IENZ_A_1841182_SM7385.zip › 14 Merlo et al Figure S5.pdf]

Figure S6

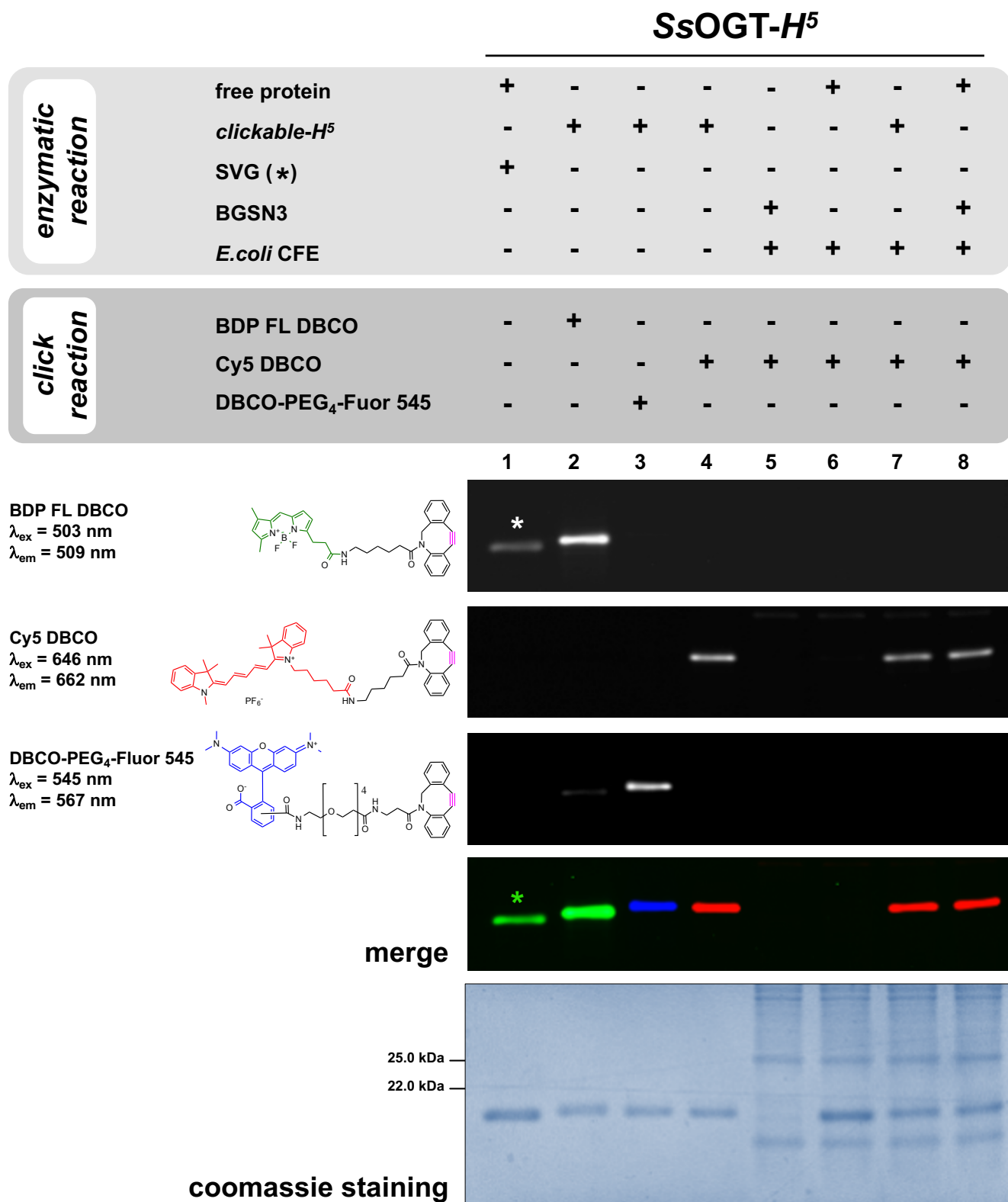

Supplement: Supplemental Material [file IENZ_A_1841182_SM7385.zip › 15 Merlo et al Figure S6.pdf]

Figure S7

A

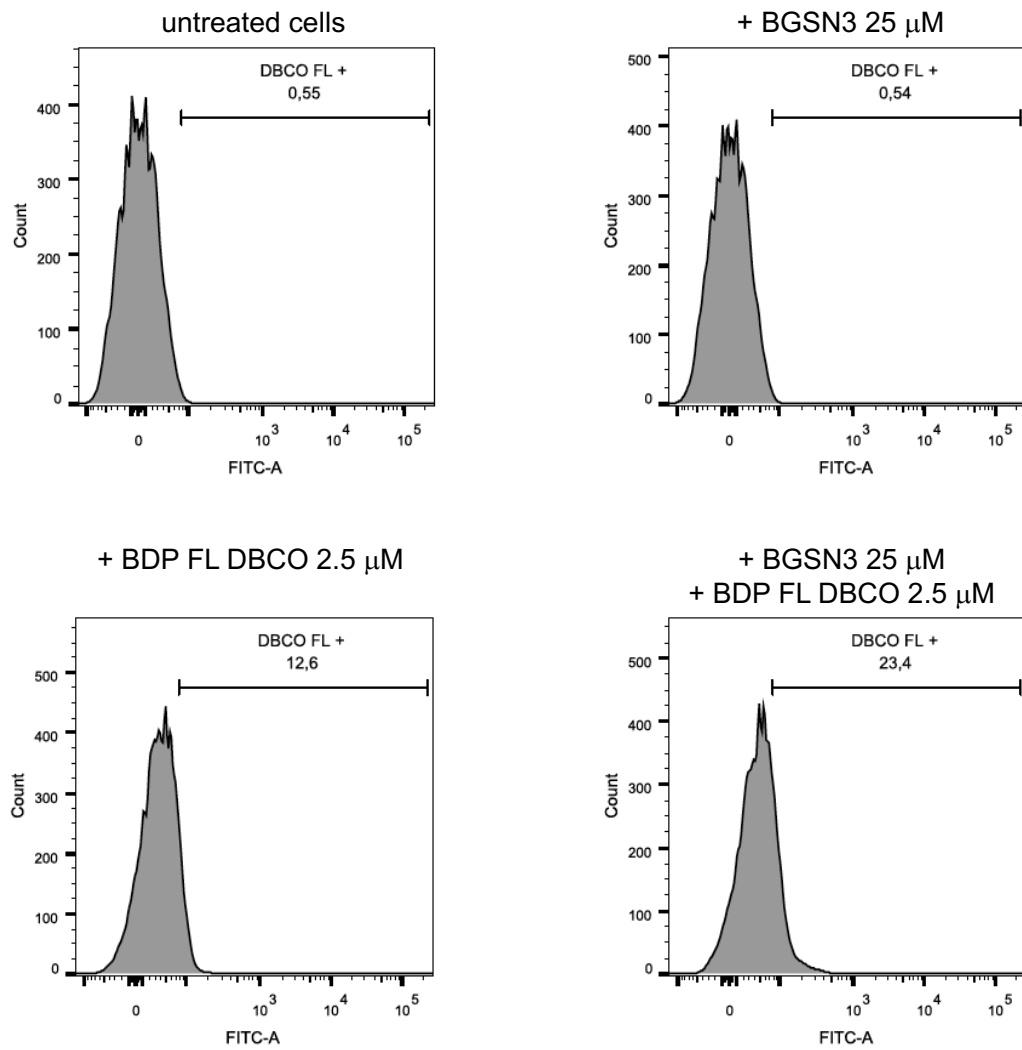

B

|                       |   |   |   |
|-----------------------|---|---|---|
| SNAP-tag <sup>®</sup> | + | - | - |
| ABLE C / pQE-SNAP     |   | + | + |
| BGSN3                 | - | + | - |

|     |   |   |   |
|-----|---|---|---|
| SVG | + | + | + |
|-----|---|---|---|

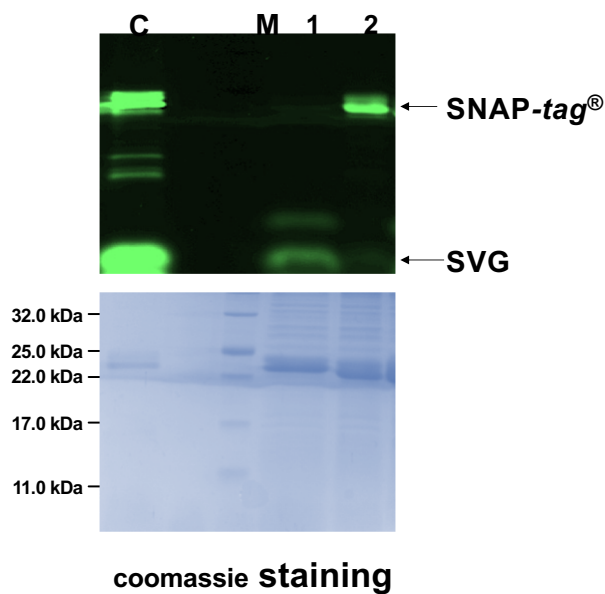

Supplement: Supplemental Material [file IENZ_A_1841182_SM7385.zip › 16 Merlo et al Figure S7.pdf]
